# Satisfaction With Virtual Consultation Services and Associated Factors Among Health Care Providers in Government Health Clinics in Selangor, Malaysia

**DOI:** 10.7759/cureus.70591

**Published:** 2024-10-01

**Authors:** Ihsan Abdul Razak, Faridah Kusnin, Surianti Sukeri

**Affiliations:** 1 Community Medicine Department, Universiti Sains Malaysia School of Medical Sciences, Kubang Kerian, MYS; 2 Primary Health Care Unit, Selangor State Health Department, Shah Alam, MYS

**Keywords:** extended technology acceptance model, health care providers (hcps), teleconsultation, telehealth, telemedicine, user satisfaction, virtual consultation

## Abstract

Background: In Malaysia, virtual consultation services (VCSs) have been implemented by the Ministry of Health (MOH) since 2019 to complement current healthcare service delivery in government health clinics. Although the services have been proven to benefit patients, little is known about the satisfaction of healthcare providers (HCPs) who run the services. This study utilizes the extended Technology Acceptance Model to evaluate the satisfaction of HCPs with VCS and identify associated factors to further improve these services.

Objectives: In this study, we aimed to determine the proportion of HCPs who were satisfied with the VCS and identify factors contributing to this satisfaction.

Methodology: A cross-sectional study was conducted among 42 government health clinics in Selangor, Malaysia, using an online survey comprising a validated questionnaire of the extended Technology Acceptance Model. A total of 137 HCPs from various categories responded to the online survey. Data were analyzed using descriptive statistics and logistic regression.

Result: The majority of the respondents (72.3%) were satisfied with the VCSs, with a mean score of 14.47 ± 3.391. Two factors were found to be significantly associated with HCPs’ satisfaction: perceived usefulness (adjusted odds ratio = 9.396; 95% confidence interval = 3.196-27.625) and behavioral intention (adjusted odds ratio = 8.311; 95% confidence interval = 2.494-27.694).

Conclusion: Our results indicated a moderately high percentage of HCPs in government health clinics of Selangor who were satisfied with VCS (72.3%). Perceived usefulness and behavioral intention of HCPs were two strong determinants of satisfaction with VCS. Therefore, efforts should be directed toward improving satisfaction with VCS by addressing these factors to ensure the sustainability of these services to best benefit patients.

## Introduction

Telemedicine, as an information and communications technology, uses digital communication between patients and health care providers (HCPs) to limit personal interaction [1]. Telemedicine uses telecommunication technology to diagnose and treat patients from a distance [2]. Since the COVID-19 pandemic began in March 2020, the use of telemedicine, as one of the most efficient and cost-effective strategies to provide health services globally, has been facilitated by movement restriction orders, social distancing, and quarantine to slow the rapid transmission of the disease [1,3,4]. In Malaysia, telemedicine was introduced in the late 1990s primarily to enhance accessibility to health services for patients living in rural or distant areas, beginning with the introduction of the first Malaysian Telemedicine Blueprint by the Ministry of Health (MOH) in 1997 [5,6]. In 2019, the MOH initiated the Virtual Clinic Program as a proof of concept in five selected government health clinics. With the emergence of COVID-19, the program expanded throughout the country and was later rebranded as virtual consultation services (VCSs) to provide a larger scope of patient services in line with the MOH’s goal of national health digitalization [7]. To date, 270 government health clinics provide VCS across the country.

Satisfaction, such as that of patients or providers, can be defined as the sum of one’s feelings or attitudes toward a variety of factors affecting the situation [8]. Satisfaction has been a major area of research on telemedicine use globally [9]. Studies in Qatar, India, the USA, and China revealed that medical practitioners are generally satisfied with telemedicine despite numerous barriers and challenges [10-13]. In Malaysia, few studies have been conducted to assess HCP satisfaction with telemedicine, particularly in the primary care setting. However, studies from Bashshur et al., Chi et al., Thong et al., and Zailani et al. showed that telemedicine plays a major role in addressing limitations faced by primary health clinics and is able to reduce congestion in hospitals [5,14-16].

The Technology Acceptance Model (TAM) is the most widely employed model for assessing technology utilization and has been empirically tested in many different areas and study settings [17]. The model had been duplicated, expanded upon, and elaborated in several studies over the years to evaluate numerous technological applications across various disciplines [18,19]. The extended TAM maintains the original domains of perceived usefulness (PU), perceived ease of use (PEOU), behavioral intention (BI), and actual telemedicine use (ATU) but adds the reflective domain of user satisfaction (US), as suggested by some authors [8,20,21]. PU is defined as physicians’ belief that telemedicine will be beneficial, expedite care delivery, save costs, and improve quality health outcomes and documentation; PEOU is defined as how simple physicians believe it will be for them to learn and use telemedicine; BI is defined as physicians’ intention to either reject or accept telemedicine services; ATU is defined as the actual implementation and use of telemedicine services in public health clinics; and US is defined as physicians’ level of satisfaction in using telemedicine services.

Since the implementation of virtual clinics in primary care in Malaysia before the COVID-19 pandemic, the acceptance of HCPs involved in implementing VCS has not been adequately assessed, and studies addressing this issue are lacking [6,22]. In view of the rapid adoption of digital health technology for patient care in Malaysia, there is an urgent need to assess the satisfaction of HCPs to ensure acceptance and delivery of high-quality clinical services, particularly in government health facilities. Therefore, the primary aim of this study is to evaluate the satisfaction of HCPs in using VCS in government health clinics and identify any associated factors.

## Materials and methods

Study design and location

This was a cross-sectional study conducted from October 2022 to June 2023. Selangor was chosen for the study location because it is the most populated state in Malaysia with the highest number of government health clinics that have adopted VCS. To date, there are 42 government health clinics under the supervision of the Selangor State Health Department. 

Study participants

The study participants were all HCPs who were involved with VCS in government health clinics in Selangor, comprising family medicine specialists (FMS), medical officers, pharmacists, dietitians, nutritionists, occupational therapists, and physiotherapists. The inclusion criteria were as follows: able to understand the English language and had at least one experience with using VCS in government health clinics in Selangor. All visiting FMS, pharmacist, dietitians, nutritionist, occupational therapists, and physiotherapists were excluded from the study. 

Sampling and study size

Convenience sampling was applied for this study. Although it is acknowledged that this sampling method results in the inability to apply the study findings to the general population in Malaysia, it was chosen due to the limited number of respondents who fulfilled the study criteria. Study participants were recruited with the help of a co-researcher who provided a list of potential government health clinics. Posters containing a QR code of the research questionnaire were posted on the notice boards of health clinics involved with VCS to disseminate information about the study. The calculated sample size was 135, with level of confidence of 95%, considering an anticipated dropout rate of 20%. A total of 215 respondents participated in the online survey; however, 95 respondents were excluded, as they did not fulfill the study criteria, and three respondents did not consent to participate in the study. Therefore, the total sample size to be analyzed was 137 participants. 

Instrument and variables

This study used a proforma (Appendices section) and a validated (unnamed) extended TAM questionnaire (Appendices section) adapted from Kissi et al. [21]. The validated questionnaire’s Cronbach alpha values were above the cutoff point of 0.70, ranging from 0.883 to 0.845. The 20-item questionnaire comprised five domains: PU (four items), PEOU (four items), BI (four items), ATU (four items), and US (four items), with responses graded on a five-point Likert scale. The first four domains (PU, PEOU, BI, and ATU) represent the independent variables of this study, and the last domain (US) represents the dependent variable. The questionnaire is scored by adding all items’ scores under each domain to give the total score. If the total score of each domain is above average (i.e., >12), it indicates positive responses from the participants; if the total score is below average (i.e., ≤12), it indicates unfavorable responses (except for the PEOU domain, with items scored in reverse). Similar to the study by Kissi et al. [21], who granted permission to use the questionnaire, this study’s questionnaire was distributed among HCPs experienced in using VCS.

Data collection procedures

Data collection was carried out online with a link to the survey distributed to email addresses and WhatsApp numbers of potential respondents by liaison officers at each clinic. Informed consent was obtained from each respondent prior to them filling out the online questionnaire. The approximate time to fill out the Google form (both the proforma and questionnaire) was five minutes. When a poor response was observed, a reminder was given to the respective liaison officers to encourage more participation from the HCPs. Ethical approval was obtained from Medical Research and Ethics Committee (MREC) of the MOH (NMRR ID-22-02853-8JQ) and Human Research Ethics Committee (HREC) of the Universiti Sains Malaysia (USM/JEPeM/22110723) prior to data collection.

Statistical analysis

The data derived from the Google form were entered into MS Excel (Microsoft Corporation, Redmond, Washington, United States)and then exported into IBM SPSS Statistics for Windows, Version 27 (Released 2020; IBM Corp., Armonk, New York, United States). Descriptive statistics were used to summarize the sociodemographic characteristics of the subjects and calculate the mean scores of the five TAM domains, and the proportion of HCPs who were satisfied with the VCS. The scores derived from the TAM domains were recoded and categorized. For the PU, BI, and ATU domains, a total score of >12 was recoded as “1 = above average,” and a total score of ≤12 was recoded as “0 = below average.” For the PEOU domain, a total score of ≤12 was recoded as “1 = above average,” and a total score of >12 was recoded as “0 = below average.” For the US domain, a total score of >12 was recoded as “1 = Satisfied,” and a total score of ≤12 was recorded as “0 = Not satisfied.” Simple and multiple logistic regression analyses were carried out for each independent variable, with results presented in terms of crude odds ratio (OR) and adjusted OR. The independent variables comprised age, gender, level of education, previous experience with VCS, and the four TAM domains PU, PEOU, BI, and ATU. The fifth TAM domain, US, served as the binary dependent variable.

## Results

Participants characteristics

Demographic characteristics of the study participants are summarized in Table 1. The majority of the respondents were <40 years of age (83.2%), and the mean age was 36.6 ± 4.6 years. Most of the respondents were female (83.9%), degree holders (83.2%), and medical officers (51.1%). The majority of the respondents did not have prior experience with VCS using the BookDoc or Skype for Business platforms (82.5%). 

**Table 1:** Demographic characteristics of health care providers (n = 137) SD: Standard deviation

Variable	Mean ± SD	n	(%)
Age (years)	36.6 ± 4.6		
<40		114	83.2
≥40		23	16.8
Gender			
Male		22	16.1
Female		115	83.9
Level of education			
Bachelor’s degree		114	83.2
Postgraduate degree		23	16.8
Job category			
Family medicine specialist		18	13.1
Medical officer		70	51.1
Pharmacist		11	8.0
Dietitian		18	13.1
Nutritionist		12	8.8
Occupational therapist		7	5.1
Physiotherapist		1	0.7
Previous experience with other virtual consultation services (e.g., BookDoc or Skype for Business)?			
Yes		24	17.5
No		113	82.5

Proportion of HCPs who are satisfied with the VCS

The scores (means ± standard deviations) for the PU, PEOU, BI, ATU, and US domains are presented in Table 2. The majority of the respondents perceived VCS as useful (73%; PU = 14.2 ± 3.6), had a high acceptance toward VCS (78.8%; BI = 14.79 ± 3.4), and used VCS regularly (75.9%; ATU = 14.5 ± 3.6). However, most of the respondents perceived VCS as difficult to use (56.9%; PEOU = 11.8 ± 2.4). Overall, the HCPs were satisfied with VCS in Selangor (72.3%), with a mean US score of 14.47 ± 3.4. 

**Table 2:** Scores of perceived usefulness, perceived ease of use, behavioral intention, actual telemedicine use, and user satisfaction domains toward virtual consultation services (n = 137) SD: Standard deviation; n: sample size

Domain	Mean ± SD	Above average		Below average	
		n	%	n	%
Perceived usefulness (PU)	14.2 ± 3.6	100	73.0	37	27.0
Perceived ease of use (PEOU)	11.8 ± 2.4	59	43.1	78	56.9
Behavioral intention (BI)	14.8 ± 3.3	108	78.8	29	21.2
Actual telemedicine use (ATU)	14.6 ± 3.6	104	75.9	33	24.1
Domain	Mean ± SD	Satisfied		Not satisfied	
		n	%	n	%
User satisfaction (US)	14.5 ± 3.4	99	72.3	38	27.7

Factors associated with HCPs’ satisfaction toward VCS

Based on the results of the simple logistic regression analysis shown in Table 3, all domain variables were significantly associated with HCP satisfaction (p < 0.05). However, based on the results of the subsequent multiple logistic regression shown in Table 4, only PU and BI were significantly associated with HCP satisfaction (p ≤ 0.001).

**Table 3:** Factors associated with health care provider’s satisfaction toward virtual consultation services in government health clinics in Selangor (n = 137) ᵅSimple logistic regression; n: sample size; OR: odds ratio; CI: confidence interval

Variable	Crude OR (95% CI)	p-valueᵅ
Age (years)		
<40	1	
≥40	1.467 (0.503-4.277)	0.483
Gender		
Female	1	
Male	0.791 (0.295-2.122)	0.641
Level of education		
Bachelor’s degree	1	
Postgraduate degree	2.019 (0.639-6.379)	0.231
Previous experience with other virtual consultation services		
No	1	
Yes	1.567 (0.540-4.549)	0.408
Perceived usefulness (PU)		
Not useful	1	
Useful	21.845 (8.377-56.971)	<0.001
Perceived ease of use (PEOU)		
Difficult to use	1	
Easy to use	2.744 (1.205-6.246)	0.016
Behavioral intention (BI)		
Low acceptance	1	
High acceptance	23.767 (8.308-67.987)	<0.001
Actual telecommunication use (ATU)		
Low usage	1	
High usage	13.647 (5.427-34.318)	<0.001

For the multiple logistic regression analysis, the forward LR method was applied. The two-way interaction between these two variables was checked, and no significant interaction was found. The model did not exhibit any multicollinearity, as the variance inflation factor was <10 and the tolerance was >0.1. In terms of the fitness of the model, the Hosmer-Lemeshow test was not significant (p-value = 0.267), suggesting the good fit of the model. As indicated by the classification table, the model correctly predicted 83.2% of cases. The area under the receiver operating characteristics (ROC) curve was 0.86 (95% CI: 0.780, 0.940), indicating that the model could accurately discriminate 86% of cases, and the model was fitted appropriately.

Interpretation of final model

HCPs who perceived VCS as useful were 9.396 times more likely to be satisfied with VCS (95% CI: 3.196-27.625; p < 0.001) compared to those who perceived VCS as not useful when adjusted for BI (Table 4). HCPs with high acceptance toward VCS were 8.311 times more likely to be satisfied with VCS (95% CI: 2.494-27.694; p < 0.001) compared with those with low acceptance toward VCS when adjusted for PU. These findings implied that HCPs who perceived VCS as useful and had high acceptance toward VCS were likely to be highly satisfied with VCS. 

**Table 4:** Factors associated with health care provider’s satisfaction toward virtual consultation services in government health clinics in Selangor (n = 137) ᵇMultiple logistic regression; n: sample size; OR: odds ratio; CI: confidence interval

Variable	β	Adjusted OR (95% CI)	p-valueᵇ
Perceived usefulness (PU)	2.240		
Not useful		1	
Useful		9.396 (3.196-27.625)	<0.001
Behavioral intention (BI)	2.118		
Low acceptance		1	
High acceptance		8.311 (2.494-27.694)	0.001

## Discussion

Most of the HCPs working in government health clinics in Selangor (72.3%) were satisfied with VCS. This result agreed with studies conducted previously in Malaysia by Thong et al. and Ibrahim et al., who reported that more than 70% of doctors were satisfied with telemedicine [15,23]. Our results also agreed well with studies from other countries such as China, Korea, Saudi Arabia, and Qatar, which reported that the majority of health professionals were satisfied with their telehealth platforms, were willing to continue using them, and would recommend telemedicine to patients [13,22,24-26]. However, most of these studies were conducted at the height of the COVID-19 pandemic. Therefore, additional studies are needed to assess the satisfaction level of HCPs, as their needs and challenges related to the use of VCS may be different post-pandemic.

Sociodemographic factors of HCP’s satisfaction with VCS

None of the sociodemographic variables in this study demonstrate associations with satisfaction toward VCS. For example, age and gender did not significantly influence HCP satisfaction with VCS (p > 0.05). Other studies also reported similar findings, in which there were no differences in overall satisfaction toward virtual consultation based on age or gender [27,28]. Individual attitudes toward technology can vary greatly, regardless of age or gender. In other words, the adoption of technology is often more about personal inclination than age or gender. One study found that personal innovativeness in technology was a stronger predictor of virtual consultation adoption than demographic variables such as age and gender [29]. Good training and technical support can also greatly enhance satisfaction with VCS, as proposed by Edirippulige and Armfield, who found that effective training significantly improved telemedicine usability and satisfaction, regardless of age and gender [30]. 

Education level also did not influence HCPs’ satisfaction with VCS (p > 0.05) in Selangor. Although many studies investigating the effect of education level on satisfaction with VCS have focused on patients rather than providers, many authors proposed that user qualification is an important demographic characteristic that influences one’s readiness to utilize virtual consultation, which could lead to higher satisfaction [31-33]. All respondents in this study were from professional categories with a minimum qualification of a bachelor’s degree (n = 114; 83.25%). There was no marked difference in terms of satisfaction level toward VCS based on education, possibly because of the narrow gap in education level among the respondents. Furthermore, medical professionals undergo rigorous and standardized medical training, which includes the use of telemedicine technologies regardless of educational level [30]. 

The majority of the respondents in this study had no prior experience in using VCS (n = 113; 82.5%). Only 24 (17.5%) respondents had experience in using previous VCS such as BookDoc and Skype for Business. These two applications were the earlier platforms used for VCS before the current application of the Google Meet platform, which government health clinics introduced in 2021. The findings of this study do not support an association between previous experience with VCS with HCP satisfaction, despite many studies suggesting a strong relationship due to familiarity with the technology [22,31,34,35]. It has been postulated that effective education and training provided by the MOH are key factors of HCP satisfaction with the current platform of VCS, irrespective of prior experience [7]. In addition, telemedicine technology has evolved rapidly, resulting in more efficient and user-friendly platforms such as Google Meet, with improved functionality and ease of use. These improvements may lead to a better user experience, even for providers who are new to telemedicine.

Variables from extended TAM

Based on the original theory of the TAM, PU and PEOU are strong determinants of BI [36]. Examining the effects of both PU and PEOU on HCP satisfaction with VCS may help researchers to understand the usefulness of the current VCS, as well as the burden and complexity of using the current system, so that effective measures can be done to increase HCP satisfaction. In this study, PU was found to be highly associated with US (adjusted OR = 9.396; 95% CI = 3.196-27.625), which corresponds with other similar studies that concluded that PU is positively associated with US [22,37,38]. The direct correlation between PU and US among HCPs may be explained by the high quality of the current virtual consultation system, which is comparable with face-to-face sessions between HCPs and patients. More importantly, HCPs are able to achieve the intended clinical outcome for their patients through these sessions [25-27]. 

Our results showed that PEOU was not significantly associated with US (p > 0.05). Studies by Wang and Nguyen et al. reported that PEOU had a significant impact on US [22,37]. In contrast, a study in Korea found that PEOU had no significant direct effect on US but may indirectly affect US through PU [38]. PEOU is more related to the usability and technical aspects of the VCS system. As certain government health clinics may have different IT equipment and internet connectivity levels depending on budget and location, the usability of the VCS system may differ among clinics. Therefore, it is only logical that PEOU does not correlate with US.

The current study demonstrated that BI directly impacted HCPs’ satisfaction with VCS (adjusted OR = 8.311; 95% CI = 2.494-27.694), despite many authors suggesting an indirect relationship between BI and US [21,39,40]. One possible explanation is that HCPs from government health clinics in Selangor had a positive intention to adopt and use VCS because they provided a reliable alternative to continue serving patients during the COVID-19 pandemic when movement was restricted. Furthermore, this intention persisted because VCS are still relevant and can reduce current workloads post-pandemic. 

Our results also showed that ATU was not significantly associated with US (p > 0.05). This finding contradicts established extended TAM theory, as evidenced by many studies that reported a positive effect of ATU on US [21,40,41]. Our best explanation for this difference is that actual VCS used may be highly influenced by existing workload in government health clinics. Although HCPs had the intention to use VCS for their perceived benefits, the number of case referrals to government health clinics was not reduced, especially post-pandemic, and did not correspond with the existing number of clinic staff. Although VCS are usually used for stable patients who only require routine follow-up and continuation of regular medications, certain clinics still require FMS to run these services owing to their advanced knowledge and skills and the limited number of medical officers. Moreover, many clinics still depend on visiting staff to ensure continuity of their services, such as rehabilitation therapy and nutrition counselling, thus limiting the actual usage of VCS in government health clinics.

Study limitations

The main limitation of this study was its use of convenience sampling. Although this sampling method allowed for the efficient collection of data from HCPs in Selangor, Malaysia, the study sample was not representative of all HCPs in the region. Therefore, sampling bias may have occurred, in which the findings cannot be generalized to other HCPs working in the rest of Malaysia. Moreover, Selangor is a heavily urbanized state with different levels of infrastructure, internet connectivity, availability of IT equipment, and resource pools of human capital with IT skills, as compared to other states, further limiting the generalizability of our findings. In addition, the study was conducted with a limited sample size, which may not fully capture the diverse experiences and views of all HCPs in the state’s government clinics. Furthermore, some factors that could influence provider satisfaction with virtual consultations were not considered in this study, such as technical proficiency, workload, or support from clinic management. Finally, this study adopted a questionnaire created in another country, which is not locally produced. A validated and translated questionnaire based on local setting would be more appropriate and give more accurate findings.

## Conclusions

The majority of HCPs were satisfied with current VCS in Selangor, Malaysia, with PU and BI being strong predictors of HCP satisfaction regardless of demographic background. On the other hand, it is important to note that simply making VCS easier to use and demanding increased VCS usage will not influence HCPs’ satisfaction with VCS. Based on our results, we recommend making the services more useful and valuable for both patients and HCP by providing more staff, expanding the services to other platforms to enable more meaningful and interactive VCS sessions based on HCP preferences, ensuring flexible use of VCS based on the needs of HCPs and patients, offering high-quality technical support, and encouraging more training and research activities in VCS.

For future studies, our first recommendation is to use other methods of assessment, including qualitative inquiry, to further understand factors related to HCPs’ satisfaction with VCS. Secondly, other variables should be included, such as job categories, number of years in practice, frequency of VCS use, and place of practice. Thirdly, random sampling should be applied to include HCPs from all over the country who work in government health clinics. Fourthly, other potential VCS methods should be explored to provide HCPs with more options and enhance their satisfaction with VCS. Finally, satisfaction also should be evaluated from patients’ perspectives to further understand the usability of VCS.

## Figures and Tables

**Figure 1:**
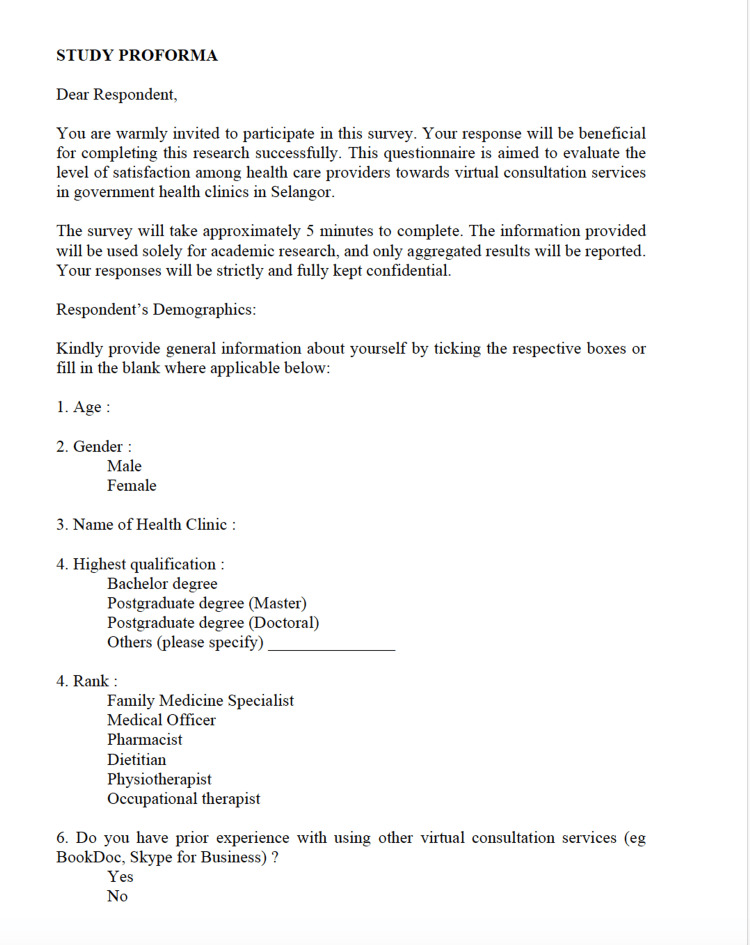
Study proforma

**Figure 2:**
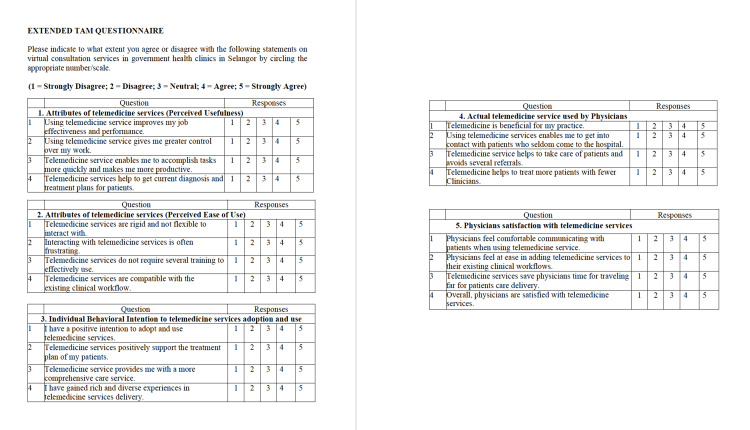
Extended Technology Acceptance Model questionnaire The extended Technology Acceptance Model questionnaire was adapted from the original article by Kissi et al. [21]. Permission to use the questionnaire was obtained from the original author

## References

[1] Bokolo AJ (2021). Exploring the adoption of telemedicine and virtual software for care of outpatients during and after COVID-19 pandemic. Ir J Med Sci.

[2] NEJM Catalyst (2018). What is telehealth?. N Engl J Med.

[3] Althumairi A, AlHabib AF, Alumran A, Alakrawi Z (2022). Healthcare providers' satisfaction with implementation of telemedicine in ambulatory care during COVID-19. Healthcare (Basel).

[4] Ng SW, Hwong WY, Husin M, Ab Rahman N, Nasir NH, Juval K, Sivasampu S (2022). Assessing the availability of teleconsultation and the extent of its use in Malaysian public primary care clinics: cross-sectional study. JMIR Form Res.

[5] Chi OW, Tan E, Yee WS, Ming WT, Ian SC, Mohd Esa NY (2022). Characteristic of patients attending virtual clinics and their feedbacks during Covid-19 pandemic: a Malaysian urban private health care Eeperience. Annals of Community Medicine and Primary Health Care.

[6] Maarop N, Win K, Masrom M, Hazara Singh S.S (2011). Exploring factors that affect teleconsultation adoption: in the case of Malaysia. https://ro.uow.edu.au/cgi/viewcontent.cgi?referer=&httpsredir=1&article=11152&context=infopapers.

[7] MOH (2022) (Ed, Division Division, F. H. D.) Ministry of Health, Malaysia Malaysia (2023). Pelan Strategik Pendigitalan Kementerian Kesihatan Malaysia 2021-2025. https://www3.moh.gov.my/moh/resources/Penerbitan/Penerbitan%20Utama/PELAN%20STRATEGIK%20PENDIGITALAN%20KKM%202021-2025/mobile/index.html#p=2.

[8] Legris P, Ingham J, Collerette P (2003). Why do people use information technology? A critical review of the technology acceptance model. Information & Management.

[9] Whitten P, Love B (2005). Patient and provider satisfaction with the use of telemedicine: overview and rationale for cautious enthusiasm. J Postgrad Med.

[10] Acharya RV, Rai JJ (2016). Evaluation of patient and doctor perception toward the use of telemedicine in Apollo Tele Health Services, India. J Family Med Prim Care.

[11] Batsis JA, Pletcher SN, Stahl JE (2017). Telemedicine and primary care obesity management in rural areas-innovative approach for older adults?. BMC Geriatr.

[12] Khan KL, Kanani S, Nisa M (2022). Assessment of primary care physicians' perception of telemedicine use during the COVID-19 pandemic in Primary Health Care Corporation, Qatar. Cureus.

[13] Ma Q, Sun D, Tan Z (2022). Usage and perceptions of telemedicine among health care professionals in China. Int J Med Inform.

[14] Bashshur RL, Howell JD, Krupinski EA, Harms KM, Bashshur N, Doarn CR (2016). The empirical foundations of telemedicine interventions in primary care. Telemed J E Health.

[15] Thong HK, Wong DK, Gendeh HS, Saim L, Athar PP, Saim A (2021). Perception of telemedicine among medical practitioners in Malaysia during COVID-19. J Med Life.

[16] Zailani S, Gilani MS, Nikbin D, Iranmanesh M (2014). Determinants of telemedicine acceptance in selected public hospitals in Malaysia: clinical perspective. J Med Syst.

[17] Husin M, Rahman NA, Bujang MA, Ng SW, Juval K, Hwong WY, Sivasampu S (2022). Translation and validation of the questionnaire on acceptance to telemedicine from the technology acceptance model (TAM) for use in Malaysia. Biomed Res Int.

[18] Lee Y, Kozar K, Larsen K (2003). The technology acceptance model: past, present, and future. Commun Assoc Inf Syst.

[19] Venkatesh V, Morris MG, Davis GB, Davis FD (2003). User acceptance of information technology: toward a unified view. Manag Inf Syst Q.

[20] Akritidi D, Gallos P, Koufi V, Malamateniou F (2022). Using an extended technology acceptance model to evaluate digital health services. Stud Health Technol Inform.

[21] Kissi J, Dai B, Dogbe CS, Banahene J, Ernest O (2020). Predictive factors of physicians' satisfaction with telemedicine services acceptance. Health Informatics J.

[22] Nguyen M, Waller M, Pandya A, Portnoy J (2020). A review of patient and provider satisfaction with telemedicine. Curr Allergy Asthma Rep.

[23] Ibrahim MI, Phing C, Palaian S (2010). Evaluation of knowledge and perception of Malaysian health professionals about telemedicine. J Clin Diagn Res.

[24] Alsaleh MM, Watzlaf VJ, DeAlmeida DR, Saptono A (2021). Evaluation of a telehealth application (Sehha) used during the Covid-19 pandemic in Saudi Arabia: provider experience and satisfaction. Perspect Health Inf Manag.

[25] Neshnash M, Metwally N, Ismail M (2022). Satisfaction of primary care physicians towards initiation of phone consultations during the COVID-19 pandemic management in Qatar: a cross-sectional study. BMC Prim Care.

[26] Park HY, Kwon YM, Jun HR, Jung SE, Kwon SY (2021). Satisfaction survey of patients and medical staff for telephone-based telemedicine during hospital closing due to COVID-19 transmission. Telemed J E Health.

[27] Pinar U, Anract J, Perrot O (2021). Preliminary assessment of patient and physician satisfaction with the use of teleconsultation in urology during the COVID-19 pandemic. World J Urol.

[28] Wang YC, Ganzorig B, Wu CC (2018). Patient satisfaction with dermatology teleconsultation by using MedX. Comput Methods Programs Biomed.

[29] Paré G, Leaver C, Bourget C (2018). Diffusion of the digital health self-tracking movement in Canada: results of a national survey. J Med Internet Res.

[30] Edirippulige S, Armfield NR (2017). Education and training to support the use of clinical telehealth: a review of the literature. J Telemed Telecare.

[31] Law T, Cronin C, Schuller K, Jing X, Bolon D, Phillips B (2019). Conceptual framework to evaluate health care professionals' satisfaction in utilizing telemedicine. J Am Osteopath Assoc.

[32] Scott Kruse C, Karem P, Shifflett K, Vegi L, Ravi K, Brooks M (2018). Evaluating barriers to adopting telemedicine worldwide: a systematic review. J Telemed Telecare.

[33] Yu-Tong T, Yan Z, Zhen L, Bing X, Qing-Yun C (2022). Telehealth readiness and its influencing factors among Chinese clinical nurses: a cross-sectional study. Nurse Educ Pract.

[34] Aldamkh BA, A Alanzan AA, AlAbdulsalam AA, Baraja MA (2022). Status of telemedicine use and physician satisfaction at public health care centers at King Abdulaziz Medical Center, Riyadh. Int J Med Res Health Sci.

[35] Guma J, Klimowich K, Pan J, Collins P, Cooley D (2021). Physician perceptions of stress and telemedicine. Osteopath Fam Physician.

[36] Davis FD (1989). Perceived usefulness, perceived ease of use, and user acceptance of information technology. MIS.

[37] Wang J, Chao Y (2022). Factors influencing continuous intention to use telemedicine after the COVID-19 pandemic in China: an extended technology acceptance model. Open J Soc Sci.

[38] Kim D, Chang H (2007). Key functional characteristics in designing and operating health information websites for user satisfaction: an application of the extended technology acceptance model. Int J Med Inform.

[39] Chao CM (2019). Factors determining the behavioral intention to use mobile learning: an application and extension of the UTAUT Model. Front Psychol.

[40] Sabbir MM, Taufique KM, Nomi M (2021). Telemedicine acceptance during the COVID-19 pandemic: user satisfaction and strategic healthcare marketing considerations. Health Mark Q.

[41] Venkatesh V, Bala H (2008). Technology acceptance model 3 and a research agenda on interventions. Decis Sci.

